# Predictive model of biliocystic communication in liver hydatid cysts using classification and regression tree analysis

**DOI:** 10.1186/1471-2482-10-16

**Published:** 2010-04-16

**Authors:** Hadj Omar El Malki, Yasser El Mejdoubi, Amine Souadka, Raouf Mohsine, Lahcen Ifrine, Redouane Abouqal, Abdelkader Belkouchi

**Affiliations:** 1 Surgery Departement "A" Ibn Sina Hospital, Rabat, Morocco; 2 Medical Center of Clinical Trials and Epidemiological Study (CRECET), Medical School, University Mohammed Vth Souissi, Rabat, Morocco; 3 Biostatical, clinical research and epidemiological laboratory (LBRCE), Medical School, University Mohammed Vth Souissi, Rabat, Morocco; 4 Medical ICU, Ibn Sina Hospital, Rabat, Morocco

## Abstract

**Background:**

Incidence of liver hydatid cyst (LHC) rupture ranged 15%-40% of all cases and most of them concern the bile duct tree. Patients with biliocystic communication (BCC) had specific clinic and therapeutic aspect. The purpose of this study was to determine witch patients with LHC may develop BCC using classification and regression tree (CART) analysis

**Methods:**

A retrospective study of 672 patients with liver hydatid cyst treated at the surgery department "A" at Ibn Sina University Hospital, Rabat Morocco. Four-teen risk factors for BCC occurrence were entered into CART analysis to build an algorithm that can predict at the best way the occurrence of BCC.

**Results:**

**I**ncidence of BCC was 24.5%. Subgroups with high risk were patients with jaundice and thick pericyst risk at 73.2% and patients with thick pericyst, with no jaundice 36.5 years and younger with no past history of LHC risk at 40.5%. Our developed CART model has sensitivity at 39.6%, specificity at 93.3%, positive predictive value at 65.6%, a negative predictive value at 82.6% and accuracy of good classification at 80.1%. Discriminating ability of the model was good 82%.

**Conclusion:**

we developed a simple classification tool to identify LHC patients with high risk BCC during a routine clinic visit (only on clinical history and examination followed by an ultrasonography). Predictive factors were based on pericyst aspect, jaundice, age, past history of liver hydatidosis and morphological Gharbi cyst aspect. We think that this classification can be useful with efficacy to direct patients at appropriated medical struct's.

## Background

*Echinococcus granulosus *is a tapeworm that resides and grows in the small bowel of dogs and other canines. It produces eggs that pass in the stool. Humans (accidental carrier) become infected through the oral route, either directly from an animal direct contact or by consuming unboiled or unwashed contaminated vegetables. In the duodenum, eggs liberate their larvae which go through the intestinal wall and migrate, via the portal system, to the hepatic gland and other organs [1,2]. Seventy-seven per cent of larvae will grow in the liver and may be able to develop a liver hydatid cyst (LHC) [3]. During cyst progression, biliary ducts pass through pericyst and loose their elasticity. Compression of biliary ducts wall lead to necrosis and fissures [3-6]. The Occurrence of biliocystic communication (BCC) is the major turning-point in the LHC evolution [5-10]. Its real incidence is not exactly precised but clinically it can range from 6,6 to 26% [3,5-12]. Some authors think that BCC can be present in more than 80% of cases [1,12,13]. This situation represent an anatomic entity characterized by different clinical manifestations depending on the size of the communication [2,6,7,11]. One of the main surgical goals of LHC treatment is to manage BCC when they occur. Recent study demonstrated that BCC was an independent predictive factor of morbidity after surgical treatment of LHC (OR = 2.27; 95% CI, 1.38-3.72) [3]. It can lead to longer hospital stays [7,14-16]. However, biliocystic communications, fortunately, can be suspected before surgery (clinically or ultrasography), during surgery (bile stained with hydatid fluid) or revealed during postoperative outcomes (bile leakage by discharge through drainage) [6,9-11,17-19]. Some authors have already tried to identify predictive factors of BCC in LHC [11-13]. The purpose of this study was to determine which patients with LHC that may developp BCC using classification and regression tree (CART) analysis.

## Methods

The records of 672 patients treated for liver hydatid cyst between January 1990 and December 2004 at the surgery department "A" at Ibn Sina University Hospital, Rabat Morocco were retrospectively reviewed. At the admission to our unit, the diagnosis of LHC was established by clinical history, clinical examination and abdominal ultrsonography for all patients [18]. Radiological investigations revealed: number, localisation, size, Gharbi's classification [20] (type I: pure fluid collection; type II: fluid collection with a split wall (floating membrane); type III: fluid collection with septa (honeycomb image); type IV: heterogeneous echographic patterns; type V: reflecting thick walls), appearance of biliary tract and presence or absence of BCC. Serological tests were not routinely used.

Patient recognized to have BCC was the ones that: 1) at the admission, showed an obstructive jaundice associated to biliary dilatation with or with out sepsis, or a stigmata of bilio-cystic communication at abdominal ultrasonography; 2) during the intervention, had bile flowing in the cavity from an orifice in the pericyst, or had cyst contents stained or infected bile even after negative cavity seek out of bilio-cystic communication; 3) at the postoperative period, had bile leakage diagnosed by a discharge through drainage, a visualization through fistulography or external bile tract drainage or through endoscopic retrograde cholangiography, or developped deep biliary infection (intrahepatic or subphrenic abscess, or generalized peritonitis) that can be diagnosed by an echo or CT-guided or non-guided imaging fine needle-aspiration, by a repeated surgery, or at the autopsy.

Non-operated Patients or missing data ones were excluded. Medical records of remained patients were analyzed according to the following parameters: Age, sex, medical history of hydatid disease, weight loss more than 10% of initial weight, main symptoms and delay of their onset, physical examination findings, abdominal ultrasonography cyst's characteristics (number of cyst: single or multiple, presence or absence of other organs involved with the disease), chest radiography, presence or absence of pre-operative complications and number of these factors (jaundice, fever: a temperature ≥38°C, dilatation of biliary tract, intra-peritoneal rupture, Budd-Chiari syndrome, intra-thoracic rupture), type of surgical procedure performed, thickness of the pericyst, associated extrahepatic biliary tract surgery, concomitant treatment of other cysts (lung, spleen, kidney and peritoneum), both postoperative mortality and morbidity, duration of stays after surgery and follow up.

Six hundred forty nine patients underwent open laparotomy. The choice between conservative method (unroofing, drainage) and radical surgery (pericystectomy and hepatectomy) was left to surgeon discretion. However, the radical approach was globally chosen when the cyst was unique, small and peripheral. The area around the cyst was covered and isolated with packs immersed in hydrogen peroxide. This precaution was taken to protect the surrounding tissues from parasite spread during cyst evacuation. Then, the cyst was incised at its most accessible part and was punctured. All content of the cyst was aspired. The germinative membrane was easily removed with forceps and the cavity was flushed with hydrogen peroxide. The pericyst was cleaned and smoothed out to remove even non apparent daughter cysts that may exist in the pericyst. Cyst was then widely deroofed by excising the projecting part of the pericyst. The residual cavity was examined to look for biliary fistulas. Visible biliary openings were sutured when they were ≥5 mm or treated by directed fistulization when they were ≥ 5 mm [7,17]. If the hydatid fluid was bile stained, with no evidence of biliary opening at a meticulous examination of the pericyst, the fistula was left alone and residual cavity was aspirated using external drainage. The management of remained cavity was left to surgeon decision (omentoplasty, capitonage, drainage) [7,12,21].

Statistical methods: Continuous variables were presented as mean value ± standard deviation or median interquartile range (IQR) and categorical variables were expressed as frequency and percentage. A cut-off 10 cm cyst's diameter was chosen for more commodity and also, to compare our data with other studies [3,11-13,16,22-24]. We have conducted an univariate association between each liable factors and the BCC occurrences with the χ^2 ^test. A Student *t-*test was used for parametric continuous variable and Mann-Whitney U-test was used for non parametric continuous variable. Significance was set at a *P *value less than 0.05.

We have conducted a univariate association between each liable factors and the BCC occurrences with univariate binary logistic regression analysis. Variable with *P *value <0.25 were selected to perform in a multivariate analyses using CART (Classification and Regression Trees) [25,26]. Risk algorithms were developed by using CART analysis. CART models were created with AnswerTree (SPSS, Chicago, IL), which performed recursive partitioning and automatic selection of optimal cut-off points for variables. To select the tree, the Gini impurity function was used with a minimal change in impurity of 0.0001. The maximal tree depth was set empirically at 4 levels, with a minimal number of 40 observations in each parent (upper) node and 20 observations in each child (lower) node. The following variables were included in the CART model: age, gender, past history of liver hydatidosis, right upper quadrant pain, jaundice, fever, abdominal mass, location of the cyst in the liver (anterior segments: segments III, IV, V, VI according to Couinaud segmental anatomy/posteriors segments: segments I, II, VII, VIII according to Couinaud segmental anatomy), diameter of the cyst, Gharbi's classification, biliary duct dilatation, lung hydatid cyst associated and pericyst aspect. Although the CART method for constructing models may be complex and most clinicians are not familiar with this method. The resulting decision trees are simple to use and are similar to algorithms used in most clinical guidelines. We calculated sensitivity (Sn), specificity (Sp), positive predictive value (PPV) and negative predictive value (NPV), accuracy of good classification and discriminating ability (the area under the ROC curve) to evaluate our developed model.

To obtain a set for reliable estimation of tree's independent predictive accuracy, we used 10-fold cross validation that split the data into approximately 10 parts. When the maximal tree was built on the entire sample, the sample was divided into 10 equal parts and each contained a similar distribution of outcome variable. The first 9 parts of the data were used to construct the largest possible tree, and the remaining 1 part was used to obtain initial estimates. The process was repeated on another 9 of 10 data parts while using a different part as the test sample until each part of the data had been held in reserve 1 time as a test sample. The results of the 10 mini-test samples were then combined and applied to the tree based on the entire sample. To assess the importance of variables not incorporated into the final tree, we examined the surrogate and competitor splits at each node of the tree. A surrogate split uses another predictor but results in similar classification of cases. Competitor splitters are variables that can be used instead of primary splitters, resulting in a tree with performance similar to the optimal tree in terms of error rates but possibly with less predictive accuracy [26]. Biliocystic communication rate in the first node at the top of the tree is used as reference. In child node, if the rate is lower than the rate in the first node, the rate is considered as low rate. If the rate in child node is near to the rate in the first node is considered as moderate rate. If the rate in child node is higher than the rate in the first node it is considered as high rate. The Statistical Package for the Social Sciences statistical software package (version 13.0 SPSS Inc, Chicago, Illinois) was used.

In our university we have an ethical committee and our study is a retrospective, no ethical approval is needed. We don't take any tissue from patients for the study and all data were in the patient's medical record.

## Results

For this study 649 patients were eligible. One hundred fifty nine patients (24.5%) had a BCC represented in 83 female (52%) and 76 male (48%). Mean age was 36.31 ± 15.32 years-old. Identification of the BCC was suspected before surgery in 60 patients, during the surgical exploration in 84 patients and the post operative period in 15 patients. Eighteen patients (11.3%) had previously undergone surgical therapy for LHC. Fourteen patients (8.8%) were asymptomatic. The most common symptom was pain on the right-upper quadrant in 120 patients (75.5%) and the most common finding on the physical examination was a palpable mass at this location in 70 patients (44%). Jaundice was seen in 41 patients (25.8%) and fever in 28 patients (17.6%). The median duration of symptoms and signs was 4 months (IQR: 2 months; 12 months).

There was no perioperative mortality. The overall morbidity was seen on 56 patients (35.2.8%) and *deep abdominal complications *[3,17] was seen on 52 patients (31.5%). Three patients developed a biliary peritonitis, cured by a second intervention. The median postoperative stay was 10 days (d) (IQR: 6 d; 20 d). All details patient's operative findings and procedures (with liver hydatid cyst having biliocystic communication) are reported in Tables 1.

**Table 1:** Cyst' characteristics, surgical procedures and operative findings on patients with liver hydatid cyst developing biliocystic communication.

Variables	Number of subject	%
Number of cysts		
One	104	65.4
Two	31	19.5
Three and more	24	15.1
Location of the cyst in the liver		
Anterior segment: III, IV, V, VI	48	30
Posterior segment: I, II, VII, VIII	111	70
Maaouni's distribution of the cyst^19^		
Cyst in the segment IV and/or I	12	7.5
Cyst in the Segment II and/or III	13	8.2
Cyst in the Segment V and/or VI	15	9.5
Cyst in the Segment VII and/or VIII	75	47
Multiple cysts	44	27.8
Diameter of the cyst		
≤10 cm	49	31
>10 cm	110	69
Gharbi's morphological type of the cyst		
Type I	18	11.3
Type II	14	8.8
Type III	75	47.2
Type IV	46	29
Type V	6	3.7
Biliary duct dilatation	25	15.7
Other Hydatid cyst outside the liver		
One abdominal organ	3	2
Abdominal hydatidosis	11	7
Lung	8	5
Surgical treatment		
Conservative	133	83.6
Radical	26	16.4
Cyst wall (pericyst)		
Soft	1	0.6
Fibrotic or calcified	158	99.4
Biliary fistula treatment		
Suture	107	67.3
Catheterisation	32	20.1
Drainage	20	12.6
Residual cavity management		
Capitonage	22	14
Omentoplasty	25	15.9
Drainage	58	36.9
Capitonnage + drainage	52	33.1
Common bile duct act	21	13.2

The mean age of patients with BCC was 36.31 ± 15.32 years-old *vs *39.91 ± 15.85 in patients with out BCC (*p *= 0.013). On univariate analysis, factors associated to the presence of BCC in patients with LHC was observed in 29.5% of men compared to 21.2% women (*p *= 0.017). Biliocystic communication occured in: 26.2% of patients with symptomatic diseases, 31.8% of patients with palpable abdominal mass, 64% of patients with jaundice and 40% of patients with fever compared to 14% (*p *= 0.018), 20.8% (*p *= 0.002), 20.2% (*p *< 0.001) and 22.7% (*p *= 0.02) of patients with none of these signs respectively. Localization of hydatid cyst with BCC was in 27.3% of patients in hepatic posterior segment *vs *19.8% patients (*p *= 0.033) with in anterior segments. According to Gharbi's cyst classification, BCC occured in 16.8% of patients with type I, 17.5% of patients with type II, 27% of patients with type III, 31.7% of patients with type IV and 15.4% of patients with type V (*p *= 0.015). The results of univariate analysis are resumed in Table 2.

**Table 2:** Results of univariate analysis concerning of biliocystic communication in liver hydatid cyst.

Variables	Number of patients with BCC (%)	Number of patients with out BCC (%)	*p*
Age (years) mean ± SD	36.31 ± 15.32	39.91 ± 15.85	0.013

Median duration of symptoms in months (IQR)	4 (2; 12)	6 (2; 12)	0.101
Sex			0.017
Male	76 (29.5)	182 (70.5)	
Female	83 (21.2)	308 (78.8)	
Past history of hepatic hydatidosis			0.071
No	141 (25.8)	405 (74.2)	
Yes	18 (17.5)	85 (82.5)	
Right upper quadrant pain			0.245
No	39 (21.4)	143 (78.6)	
Yes	120 (25.8)	345 (74.2)	
Jaundice			<0.001
No	118 (20.2)	465 (79.8)	
Yes	41 (64.1)	23 (35.9)	
Fever (temperature ≥38°C)			0.02
No	131 (22.7)	446 (77.3)	
Yes	28 (40)	42 (60)	
Epigastric pain			0.872
No	141 (24.5)	435 (75.5)	
Yes	18 (25.4)	53 (74.6)	
Clinical presentation			0.018
Asymptomatic	14 (14.9)	80 (85.1)	
Symptomatic	145 (26.2)	408 (73.8)	
Abdominal mass			0.002
No	89 (20.8)	338 (79.2)	
Yes	70 (31.8)	150 (68.2)	
Weight loss			0.499
No	137 (24)	433 (76)	
Yes	16 (28.1)	41 (71.9)	
Number of cysts			0.274
One	104 (23.3)	342 (76.7)	
Two	31 (31)	69 (69)	
Three or more	24 (24.7)	73 (75.3)	
Location of the cyst in the liver			0.033
Anterior segment: III, IV, V, VI	48 (19.8)	194 (80.2)	
Posterior segment: I, II, VII, VIII	111 (27.3)	296 (72.7)	
Maaouni's distribution of the cyst^19^			0.101
Cyst in the segment IV and/or I	12 (23.1)	40 (76.9)	
Cyst in the Segment II and/or III	13 (14.6)	76 (85.4)	
Cyst in the Segment V and/or VI	15 (20.3)	59 (79.7)	
Cyst in the Segment VII and/or VIII	75 (26.8)	205 (73.2)	
Multiple cysts	44 (28.8)	109 (71.2)	
Biliary duct dilatation			<0.001
No	131 (21.9)	467 (78.1)	
Yes	25 (65.8)	13 (34.2)	
Diameter of the cyst			0.240
≤10 cm	49 (21.8)	176 (78.2)	
>10 cm	110 (25.9)	314 (74.1)	
Gharbi's morphological type of the cyst			0.015
Type I	18 (16.8)	89 (83.2)	
Type II	14 (17.5)	66 (82.5)	
Type III	75 (27)	203 (73)	
Type IV	46 (31.7)	99 (68.3)	
Type V	6 (15.4)	33 (84.6)	
Other hydatid cysts in lungs			0.009
None	151 (23.9)	482 (76.1)	
Yes	8 (53.3)	7 (46.7)	
Cyst wall (pericyst)			<0.001
Soft	1 (0.6)	177 (99.4)	
Fibrotic or calcified	157 (33.5)	311 (66.5)	

BCC: biliocystic communication

**Figure 1:**
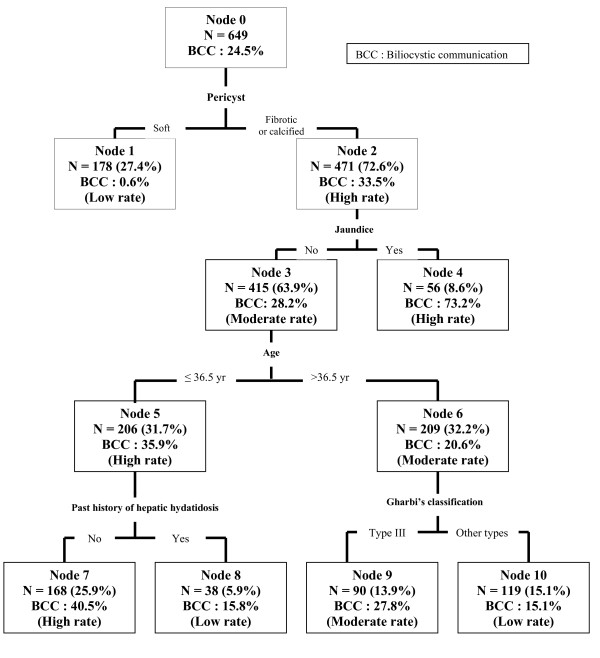
**Subgroups of patients with liver hydatid cyst identified through classification tree analysis and their risks to develop biliocystic communication**.

Using CART analysis, we successively partitioned our study population into subgroups, using the most significant predictor variables. The CART model indicated that pericyst aspect was the primary determinant for the BCC occurrence (Figure 1). Among patients that have fibrotic or calcified pericyst 33.5% had BCC, whereas only 0.6% of patients with soft pericyst had BCC. In patients with fibrotic or calcified pericyst, the next most important determinant of occurrence of BCC was jaundice: patients with jaundice had a 73% risk to develop a BCC, while anicteric patients had only a 28.2% of this risk. Concerning patients with fibrotic or calcified pericyst with no jaundice, a new split was based on the age. When patients were older than 36.5 years-old (node 6) (the age cutoff chosen by the CART algorithm) only 20.6% could developp BCC, whereas almost 35.9% of those 36.5 years or younger (node 5) had BCC. From this level each node had a different split. Among patients with fibrotic or calcified pericyst with no jaundice and 36.5 years and younger (node 5) the next determinant for BCC occurrence was their past history of hepatic hydatidosis. Patients with no past history had 40.5% of risk for having BCC *vs *15.8% risk if they had past history of hepatic hydatidosis (node 7 and 8 respectively). In the group of the patients with fibrotic or calcified pericyst with no jaundice and older than 36.5 years (node 6) the split was different and based on Gharbi's cyst type. Patients with cysts type III had 27.8% risk to develop BCC *vs *15.1% risk if they had other types (node 9 and 10 respectively).

Our developed CART model has sensitivity (Sn) at 39.6%, specificity (Sp) at 93.3%, positive predictive value (PPV) at 65.6%, negative predictive value (NPV) at 82.6% and accuracy at 80.1% (Table 3). Discriminating ability of the model was good, as shown by the area under the ROC curve (0.82).

**Table 3:** Prediction- Success cross-tab

	Presence of biliocystic communication	Absence of biliocystic communication	Total
Positive prediction	63	33	96

Negative prediction	96	457	553

Total	159	490	649

Sensitivity: 39.6%, specificity: 93.3%, positive predictive value: 65.6%, negative predictive value: 82.6% and accuracy: 80.1%.

## Discussion

In this study, which is one of the largest series in international literature of LHC [3], we tried to determine predictive factors for occurrence of BCC. Using a particular statistical methodology: classification and regression tree analysis (CART) [25], we set predictive algorithm to help us to assess patient's risk to develop BCC. Our data support that pericyst aspect (soft/fibrotic or calcified), jaundice, age, past history of hepatic hydatidosis and Gharbi's cyst type are the predictive factors of the occurrence of BCC (figure 1). This model has an accuracy of good classification at 80.1%, good discriminating ability at 82.4%, specificity at 93.3% and negative predictive value at 82.6%.

It is well established that liver hydatid cyst rupture incidence represents 15% to 40% of LHC; most of these cases concern the bilary tract (40 - 60%) [6,9,10,14,27,28]. LHC evolution is closely correlated to the relation between biliary tracts and the cyst which leads to clinical and therapeutic considerations specific to liver's location of hydatidosis [2,5,7,15,27-44]. Morbidity and mortality of LHC surgical treatment is linked to presence or absence of BCC [3,7,11,12,28,45]. Patients with BCC raise their risk two-fold to have complications (odds ratio at 2.27; 95% confidence interval: 1.38-3.72) [3]. There is no consensus concerning the terminology that should be used in hydatid cyst cases with bilio-cystic communication [6,24]. Some authors bring up only the communication size with out taking into consideration clinical features to distinguish between the two common situations (occult or frank rupture) [7,39]. Others based their description on clinical (symptoms), per-operative (bile stained fluid) and post-operative (biliary fistula) findings [11-13,28]. In current study, we have kept in mind that during LHC progression, biliary ducts pass through pericyst and loose their elasticity. Compression of biliary duct's wall leads to necrosis and fissures [3-5,13,45]. So we choosed to enlarge our inclusion criteria to simple occurrence of biliocystic communication, recognised before surgery (clinically or ultrasography or others), during surgery (bile stained of the hydatid fluid and orifice on bottom of the cavity) or during the postoperative stay (bile leakage by discharge through drainage) [6,7,9-13,17-19].

CART analysis is non-linear and non-parametric alternative to linear models for regression and classification problems (such as linear regression, logistic regression, linear discriminant analysis, linear proportional hazard models). we prefered to yse CART analysis in this study, in stead of other multivariate analysis, becquse of the following advantages: CART is robust tool that can defy violation of assumptions of continuity and normal distribution; CART can be applied to numeric and/or categorical data; CART offers dichotomous or trichotomous cutpoints, thus can provide values for outcomes that are routinely asked to be used in clinical assessments; CART facilitates the identification and interpretation of complex interactions, whereas other multivariate analysis can only handle interactions predetermined by the analyst; finally CART yields a decision tree and offers a better interpretation of the results and the judgment process. Our data assessed BCC rate at 24.5%. Pericyst aspect was the primary determinant for the BCC occurrence. When it is soft, the risk to find BCC is very low about 0.6%, whereas patients with thick (fibrotic or calcified) pericyst increase their risk to have BCC to 33.5%. These findings support results of other studies [3,5,32,38,39]. It has been demonstrated that the presence of numerous biliary duct of various sizes may exist within the pericyst [6,8,46,47]. Bile can submerge the cyst after rupture. It induces inflammation which leads to increase pericyt thickness [4,11,28,45]. None of the studies interested into predicting BCC, could assessed this factor [11-13]. Ultrasonography can be enough to inform about the thickness of cyst's wall in addition to other cyst characteristic: number, localisation, diameter and bile duct tree [6,20,32,46]. Jaundice in patients with thick pericyst was a high predictive factor of BCC occurrence risk (73.2%). These factors testify that the hydatid cyst is complicated. Jaundice in patients with LHC could be the result of biliary duct compression therefore no surgery on the biliary tree is needed after cyst treatment. It can also be the result of HCL rupture into the biliary tract, which can occur during the HCL progress. The cyst fluid, then daughters' cysts, may enter the biliary tree [3,4]. Surgical exploration of the common bile duct is recommended to extract daughters' cysts making sure of duct vacuity [6-8,27,28,31,38,39,43-45,47]. Some authors attest that raised liver function test values can be a good predictive factor of BCC occurrence [12,13]. Depending on which element of liver function was used, sensitivity ranged from 73.1 to 90.2% and specificity ranged 61.3-86% [12]. Raised leukocytes, eosinophil rate and infected bile were other predictive factors [11-13]. This biological tests require more blood samples and are the biological stigmates of the cyst rupture into bile tract. Two third of our patients (node 3 on figure 1) had a thick pericyst with no jaundice and the risk to have BCC was moderated (28.2%). CART analysis split this group on two clusters with different risks based on age cutt-off at 36.5 years. Previous studies failed to identify age as predictive factor of BCC occurrence [11-13]. Two of them assessed age as a continuous variable [11,12]. The last one transform it on categorical variable (<40 yr, ≥40 years) [13]. We chose to retain the continuous aspect of this parametric variable. The first cluster bringing together thick pericyst with no jaundice at patients with 36.5 years and younger (node 5 on figure 1) had high risk of BCC (35.9%), compared to the same cluster but with older patient, where the risk was moderate (20.6%). The last BCC determinant varied considering age of patients having thick pericyt and no jaundice. Thirty six and half years-old and younger cluster with BCC high rate (node 5) was split in final subgroups according to past history of hepatic hydatidosis. If there was no past history, patients had high risk BCC occurrence 40.5% *vs *15.8% (low risk) if there was past history of hydatidosis. This factor have never been evaluated at our knowledge. We think that patients who underwent surgery for liver hydatidosis are carefully attended by routinely ultrasound-imaging during follows-up [46]. Thus, recurrence's diagnosis can be made early before the appearance of symptoms. In older than 36.5 years cluster's, CART analysis split it into two final subgroups, based on Gharbi's morphological type of the cyst [20]: Moderate risk for type III at 27.8% (node 9) *vs *low risk at 15.1% for other types. Morphological type of the cyst Gharbi's classification, especially type III and IV (multilocular and degenerated) was assessed as predictive factor of BCC but by different results [4,11-13,15,28,45]. We agree with others authors to assess that morphological evolution of the LHC is correlated to biliocystic communication [2,4,5,7,20,45]. This result does not strongly support this idea. Our study failed to identify cyst's size as predictive factor in contrast to another study [13]. Some authors have previously report the role played by 10 cm average cyst's size as predictive factor [11,12,16,22,24]. With high cyst volume, the intracystic pressure rise leading to compression of the adjacent liver parenchyma and stretches the bile duct. Necrosis of the bile duct wall cause rupture [4,11-13,41,44,45].

Our developed model based on ultrasonography (as very sensitive and specific tool to diagnose LHC in endemic area) [18,20] and quick examination (Age, presence of jaundice and past history of liver hydatidosis) can be helpful to orientate, with a good accuracy BCC, patients to centres with high hepato-biliary experienced surgeons who may offer the best management for particular complicated cysts. Technichal staff should offer, either pre-operative ERCP or intra-operative cholangiography, intensive care unit, more surgical techniques skills and radiological interventional units. The model can be useful in patients' selection to percutaneous treatment or laproscopy [3,15,30,37]. It has a good negative predictive value 82.6% because it is more specific: 93.3% than sensitive: 39.6%.

Limitations of our study are those of all retrospective studies. We could not have the exact size for all cysts, so we convert this variable to a categorical one as done by some authors [11-13,16,22,24]. Value of pericyst's thickness was not precised. Some authors defined thickness starting from 1 mm [5]. The generalization of new generation technology of ultrasonography may provide additional benefit. We do not assess the blood sample test. Perhaps we could raise the predictive model accuracy if we considered liver function tests, blood count cells and serology; thus it would increase the cost of clinical and paraclinical evaluation. Therefore, In endemic area socio-economic possibilities are, unfortunately, very limited so the predictive model will lose its benefit/cost. This model needs an external validation. We are willing to proceed it in a current prospective study.

## Conclusions

We developed a simple classification tool to identify LHC patients with high risk of the occurrence of BCC. This classification tool identified more than 80% patients during a routine clinic visit (clinical history and ultasonography). Predictive factors were based on pericyst aspect, jaundice, age, past history of liver hydatidosis and morphological Gharbi cyst aspect. This classification will be useful to orientate patients to the appropriate medical unit.

## Abbreviations

LHC: liver hydatid cyst; BCC: biliocystic communication.

## Competing interests

The authors declare that they have no competing interests.

## Authors' contributions

Study conception and design: *HOE; *Acquisition of data: *HOE, YE, AS, RM, LI, AB; *Analysis and interpretation of data: *HOE, RA; *Drafting of manuscript: *HOE, AS*; Critical revision: *HOE, AS, RA; *All authors read and approved the final manuscript.

## Pre-publication history

The pre-publication history for this paper can be accessed here:

http://www.biomedcentral.com/1471-2482/10/16/prepub
